# X Chromosome and Autosome Dosage Responses in *Drosophila melanogaster* Heads

**DOI:** 10.1534/g3.115.017632

**Published:** 2015-04-07

**Authors:** Zhen-Xia Chen, Brian Oliver

**Affiliations:** Section of Developmental Genomics, Laboratory of Cellular and Developmental Biology, National Institute of Diabetes and Digestive and Kidney Diseases, National Institutes of Health, Bethesda, Maryland 20892

**Keywords:** segmental aneuploidy, sex determination, dosage compensation, sex chromosomes, gene expression, genetics of sex

## Abstract

X chromosome dosage compensation is required for male viability in *Drosophila*. Dosage compensation relative to autosomes is two-fold, but this is likely to be due to a combination of homeostatic gene-by-gene regulation and chromosome-wide regulation. We have baseline values for gene-by-gene dosage compensation on autosomes, but not for the X chromosome. Given the evolutionary history of sex chromosomes, these baseline values could differ. We used a series of deficiencies on the X and autosomes, along with mutations in the sex-determination gene *transformer-2*, to carefully measure the sex-independent X-chromosome response to gene dosage in adult heads by RNA sequencing. We observed modest and indistinguishable dosage compensation for both X chromosome and autosome genes, suggesting that the X chromosome is neither inherently more robust nor sensitive to dosage change.

Like in many eukaryotes, reduction of gene dosage is deleterious in *Drosophila melanogaster*. Multi-locus deletions (deficiencies) are tolerated when the extent of the deletion is modest, but when >1% of genome is hemizygous, the result is reduced viability regardless of which part of the genome is deleted (Lindsley *et al.* 1972) . The X chromosome in males is an exception to this rule.

The sex chromosome dosage problem is inherent in the evolution of sex chromosomes, an event that has occurred many times during the course of evolution (Larsson and Meller 2006; Vicoso and Charlesworth 2006; Gurbich and Bachtrog 2008; Stenberg and Larsson 2011) . The initial signal for sex determination in *Drosophila* is the number of X chromosomes (Erickson and Quintero 2007), which results in males being hemizygous for ∼20% of the genome. X chromosome dosage compensation prevents male lethality by equalizing the expression level of genes on the X chromosome in males to that in females to maintain genic balance with the autosomes in both sexes (Parisi *et al.* 2003; Gupta *et al.* 2006; Sturgill *et al.* 2007; Deng *et al.* 2011). Chromosome-wide X-chromosome dosage compensation relative to autosomes requires the *male-specific lethal (MSL)* complex (Meller and Kuroda 2002; Hamada *et al.* 2005; Deng and Meller 2006; Zhang *et al.* 2010; Georgiev *et al.* 2011; Stenberg and Larsson 2011).

It is less clear how much of the X chromosome dosage compensation is due to chromosome-wide regulation and how much is due to gene-by-gene dosage responses. For example, there are X chromosome genes that do not show MSL association but that do show dosage compensation (Philip and Stenberg 2013), and some X chromosome genes are dosage compensated very early, prior to activation of the MSL system (Lott *et al.* 2011). Finally, removal of the MSL complex components does not result in halving of X chromosome gene expression relative to autosomes (Hamada *et al.* 2005; Deng and Meller 2006; Zhang *et al.* 2010), raising the possibility that other MSL-independent mechanisms may also contribute to dosage compensation in fly somatic cells.

*A priori*, an X-chromosome-wide mechanism does not need to result in a precise two-fold upregulation of gene expression relative to autosomes, because even autosomal genes can show some dosage compensation. In *Drosophila* S2 cells, the observed autosomal dosage compensation level was roughly equal to the approximately 1.4-fold “missing” compensation following MSL complex knockdown, suggesting that there may be an essentially additive effect of MSL and general dosage compensation mechanisms (Zhang *et al.* 2010). However, other *Drosophila* cell lines show a variety of global dosage compensation levels (Lee *et al.* 2014), raising the distinct possibility that this additive effect in S2 cells is coincidence.

Several studies have reported the transcriptional effect of systematically manipulating autosomal gene dosage (Gupta *et al.* 2006; Stenberg *et al.* 2009; Stenberg and Larsson 2011; Malone *et al.* 2012). These studies suggest that the same type of gene regulation that modulates levels in transcriptional networks results in gene-by-gene dosage compensation (Malone *et al.* 2012; Chen *et al.* 2014; Lee *et al.* 2014). This gene-by-gene response also highlights the need to use a matched sample for baseline measurements of dosage compensation. Simply comparing autosomes to X chromosomes is misleading when individual genes differ in dosage responses and when the population of genes residing on the X is highly biased due to the evolution of sex chromosomes (Vicoso and Charlesworth 2006; Gurbich and Bachtrog 2008). If X chromosomes were like autosomes, gene-by-gene responses at subsets of genes should not require specialized factors to achieve dosage compensation (dosage compensation of such genes would result in overexpression), and in other subsets where gene expression collapses in response to a 50% dosage reduction even a two-fold effect might not be sufficient (Malone *et al.* 2012). However, a host of studies have shown that the X chromosome is quite different than autosomes (Vicoso and Charlesworth 2006; Gurbich and Bachtrog 2008), showing a distinct chromatin signature even in females, for example (Zhang and Oliver 2010). As another example, there is also strong gene traffic off the X in the genus, which could drive dosage-sensitive genes off the X (Sturgill *et al.* 2007; Vibranovski *et al.* 2009). The gradual acquisition of dosage compensation during sex chromosome evolution could make the X either more or less sensitive to gene dosage changes, depending on which forces are dominant. For example, a long history of chromosome-wide regulation in males could reduce endogenous gene-by-gene dosage compensation by relaxing feedback in gene regulation networks, whereas in contrast a long history of pressure to increase expression of hemizygous genes on the X before acquisition of chromosome-wide dosage compensation mechanisms could boost these same gene-by-gene regulatory mechanisms. Thus, it is unclear if X chromosome gene-by-gene dosage responses are similar to autosomal gene-by-gene responses.

In wild-type flies, sexual identity, sex-biased expression, and any special evolved features of the X chromosome are confounding factors if you are interested in what could be very subtle effects on dosage compensation. In this study, we separated these factors using autosomal and X-linked deficiencies in conjunction with sex transformation, and we further reduced confounding by restricting our analysis to heads, which show limited sex-biased expression. We performed next-generation RNA sequencing (RNA-seq) and determined expression of matched hemizygous and homozgyous genes to avoid comparing expression of X chromosomes to autosomal genes directly. We observed indistinguishable dosage responses of chromosomes X and 3L in this study, suggesting that X and autosomes have similar overall responses to dosage.

## Materials and Methods

### Samples and sequencing

We used congenic *Drosophila* strains from the European *Drosophila* deletion collection (DrosDel) project (Ryder *et al.* 2007; Cook *et al.* 2010) to assay expression changes due to gene dosage. In addition to the DrosDel lines, we examined expression in the *w^1118^* DrosDel parental line and used a *Dp(1;Y)B^S^*; *cn^1^ tra2^B^ bw^1^/CyO* and *Df(2R)trix/CyO* stocks for sex-transforming flies (Bloomington *Drosophila* Stock Center, Bloomington, IN). We also used *OreR* (gift from Elissa Lei, Laboratory of Cellular and Developmental Biology, NIDDK, NIH, Bethesda, Maryland). Flies were grown at 25° on San Diego Stock Center cornmeal media. We collected 30 5-d-old adult flies for each triplicate of 10 heads, which were dissected on dry ice and stored at −80°.

We homogenized frozen heads in 2 ml Axygen 96-well plates (Corning, Life Sciences, Union City, CA) preloaded with 200 µL 1-mm glass beads (Biospec Products Inc., Bartlesville, OK) and 200 µL RLT Buffer (Qiagen, Valencia, CA) and covered with Axygen sealing mats, in a Mini-BeadBeater 96 (Biospec Products Inc., Bartlesville, OK). We extracted total RNA with the RNeasy 96 Kit and QIAvac 96 vacuum manifold (Qiagen, Valencia, CA). We visually inspected RNA quality by gel electrophoresis detected with SYbrGold Nucleic Acid Gel Stain and we used a plate reader to quantify yield with Quant-iT RiboGreen (Life Technologies, Grand Island, NY). We used 100 ng of total RNA and the TruSeq RNA Sample Preparation V2 protocol (Illumina Inc., San Diego, CA) to make RNA-seq libraries from polyA^+^ RNA. We added 10 pg of pool 78A ERCC spike-in RNAs (Jiang *et al.* 2011) to the Elute, Prime, and Fragment Mix in the “Purify and fragment mRNA” step. We checked the library quality with the High-Sensitivity DNA Analysis Kit on a Bioanalyzer (Agilent, Santa Clara, CA) and concentration by Quant-iT PicoGreen (Life Technologies, Grand Island, NY), and performed 76nt single-end sequencing on an Illumina HiSequation 2000 running CASAVA-1.8.2/RTA 1.17.21.3/HiSeq Control Software 2.0.10.0 (Illumina Inc., San Diego, CA).

### Sequence mapping and quantification

To measure expression genome-wide, we performed single-end RNA-Seq on poly-A^+^ RNA extracted from adult female and male heads in biological triplicate. We uniquely mapped reads to FlyBase genome release 5.57 (St. Pierre *et al.* 2014) and spike-in RNAs (Jiang *et al.* 2011) that we added to the libraries to empirically determine sampling variance in complex mixtures of RNA species. We used DESeq2 (Anders *et al.* 2015) normalized read counts and cufflinks (Trapnell *et al.* 2012) fragments per kilobase per million mapped reads (FPKM) to report gene-level steady-state expression.

We downloaded GFF3 gene annotation files from NCBI (http://www.ncbi.nlm.nih.gov/), extracted CDS and exon features on existing chromosomes (excluding chromosome U and Uextra), and converted to GTF format, which is compatible with all analysis tools we used, using a custom Perl script. Some peculiar gene features were incompatible with analysis tools. We “corrected” the boundaries of 10 CDS, which were beyond that of the parent genes and changed strand information for 9 CDS (11 exons) according to the annotation of the parent genes. We also added 96 ERCC spike-in annotations in the final GTF file. We mapped RNA reads with Tophat v2.0.10 (Kim *et al.* 2013). We used parameter −g 1 to retain only uniquely mapped reads and provided the GTF format annotation.

We measured the gene expression using cufflinks v2.1.1 (Trapnell *et al.* 2012) in fragments per kilobase of transcript per million mapped reads (FPKM). We measured gene expression using HT-Seq v0.5.4p1 (Anders *et al.* 2015), which reports in read counts. We normalized read counts using DESeq2 v1.6.3 (Love *et al.* 2014) in R v3.1.1. We used coefficient of variance for genes, intergenic space, and spike-in RNAs to derive a low expression cut-off value (see below).

## Results

### Datasets

**Figure 1:**
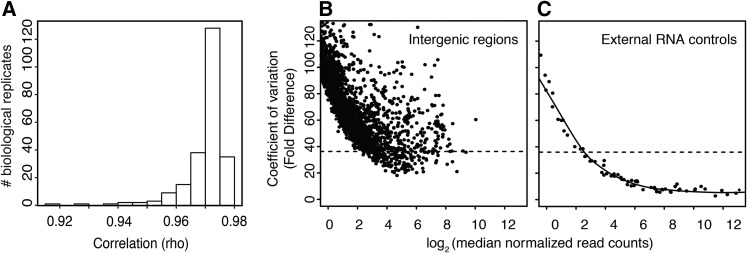
RNA-Seq libraries and expression cut-offs. (A) Correlations among biological replicate RNA-seq experiments on adult heads. (B, C) Coefficients of variance (CV) for reads mapping to intergenic space (B) and spike-in RNA controls (C). Ninety-five percent of intergenic regions have a CV above the dotted line.

We generated 264 samples for RNA-Seq profiling with a median of ∼8 million uniquely mapped reads per sample (Table 1). We failed 15 samples during library or sequencing quality checks. We also ensured that the lines still bore the originally generated deletions. Previously, we found that when a *Df/+* line did not alter gene expression in the hemizygous segment, there was no *Df* in the source stock as measured by DNA-Seq (Malone *et al.* 2012). Therefore, we scanned the gene expression ratios of each *Df/+* sample relative to +/+ controls across chromosomes 3L and X. We suspected that *Df(3L)4685* was not a deficiency based on this criterion and cytological examination also failed to show evidence of a deletion (K. Cook, personal communication). We removed nine “*Df(3L)4685*” samples as well as five samples without replicates from the analysis. These data are still useful for other purposes. Data from all 249 high-quality samples are available at the Gene Expression Omnibus (GEO) (Barrett and Edgar 2006) under accession GSE60571. Triplicates showed excellent linear correlations (Figure 1A), giving us confidence in measuring subtle differences in gene expression.

**Table 1:** Summary of samples used

Genotypes	*XX*	*XY*	*XX*; *tra2^B^/Df**^a^*	*XY*; *tra2^B^/Df*	No.*^b^*
*Df(1)ED13478/+*	3	NA*^c^*	3	NA	**6**
*Df(1)ED14021/+*	3	NA	3	NA	**6**
*Df(1)ED409/+*	3	NA	3	NA	**6**
*Df(1)ED6443/+*	3	NA	3	NA	**6**
*Df(1)ED6630/+*	3	NA	3	NA	**6**
*Df(1)ED6712/+*	3	NA	3	NA	**6**
*Df(1)ED6727/+*	3	NA	3	NA	**6**
*Df(1)ED6829/+*	3	NA	3	NA	**6**
*Df(1)ED6878/+*	3	NA	3	NA	**6**
*Df(1)ED6906/+*	3	NA	3	NA	**6**
*Df(1)ED6957/+*	3	NA	3	NA	**6**
*Df(1)ED6989/+*	3	NA	3	NA	**6**
*Df(1)ED7147/+*	0	NA	3	NA	**3***^d^*
*Df(1)ED7153/+*	3	NA	3	NA	**6**
*Df(1)ED7161/+*	3	NA	3	NA	**6**
*Df(1)ED7170/+*	3	NA	3	NA	**6**
*Df(1)ED7225^/+^*	1	NA	0	NA	**1***^e^*
*Df(1)ED7261/+*	3	NA	3	NA	**6**
*Df(1)ED7289/+*	3	NA	3	NA	**6**
*Df(1)ED7331/+*	2	NA	3	NA	**5**
*Df(1)ED7374/+*	1	NA	0	NA	**1***^e^*
*Df(1)ED7635/+*	3	NA	3	NA	**6**
*Df(3L)ED210/+*	3	3	3	0	**9**
*Df(3L)ED211/+*	3	3	3	0	**9**
*Df(3L)ED217/+*	3	3	3	0	**9**
*Df(3L)ED225/+*	3	3	3	0	**9**
*Df(3L)ED230/+*	3	3	3	0	**9**
*Df(3L)ED4287/+*	3	3	3	0	**9**
*Df(3L)ED4421/+*	3	3	3	0	**9**
*Df(3L)ED4457/+*	3	3	3	0	**9**
*Df(3L)ED4475/+*	3	3	3	0	**9**
*Df(3L)ED4543/+*	3	3	3	0	**9**
*Df(3L)ED4685/+*	3	3	3	0	**9***^f^*
*Df(3L)ED4978/+*	3	3	3	0	**9**
*w^1118^/ w^1118^* or *Y*	3	3	3	3	**12**
*w^1118^ /w^+^*	2	NA	3	NA	**5**
*OreR*	3	3	0	0	**6**
No.	**102**	**42**	**102**	**3**	**249**

a Df(2R)trix.

b Number of independent RNA-Seq profiles.

c Not applicable.

d Present in GEO, but removed from this analysis due to lack of a matching nonsex-transformed sample.

e Present in GEO, but removed from this analysis due to lack of replicates.

f Present in GEO, but removed from this analysis because we observed no dosage effect in the region of the annotated *Df*, and because no *Df* was detected in the source stock. See text.

Although RNA-seq gene expression measurements show excellent correlations, understanding dosage responses and compensation requires precision at less than two-fold differential expression. Low abundance transcripts are difficult to accurately quantify, because noise and sampling errors are more pronounced for genes with low expression (Jiang *et al.* 2011; Malone and Oliver 2011). These factors can confound dosage analysis by suggesting dosage compensation where none exists (taken to an extreme, a gene that is not expressed could be said to be perfectly dosage compensated).

To rigorously avoid overcalling compensation due to low expression, we directly modeled error profiles by comparing intergenic gene expression to genic expression. Although there may be unannotated genes in intergenic space, signal from these regions may also represent biological noise and/or DNA contamination in RNA preparations (Zhang *et al.* 2010). We also analyzed quantification variance for the synthetic spike-in RNAs with known input concentrations as determined by mass spectrophotometry (Jiang *et al.* 2011). For intergenic regions (Figure 1B), we found that 95% of intergenic regions had a coefficient of variance (CV) >36 for fold differences. At this CV value, the normalized read counts of external RNA standards fitted with a local polynomial regression was six normalized read counts (Figure 1C). Sixty-four percent of all genes were expressed at more than six, and 69% showed CVs <36. Therefore, we used six normalized read counts as a low-expression cut-off for ratiometric analysis.

### Gene expression in wild-type and sex-transformed heads

**Figure 2:**
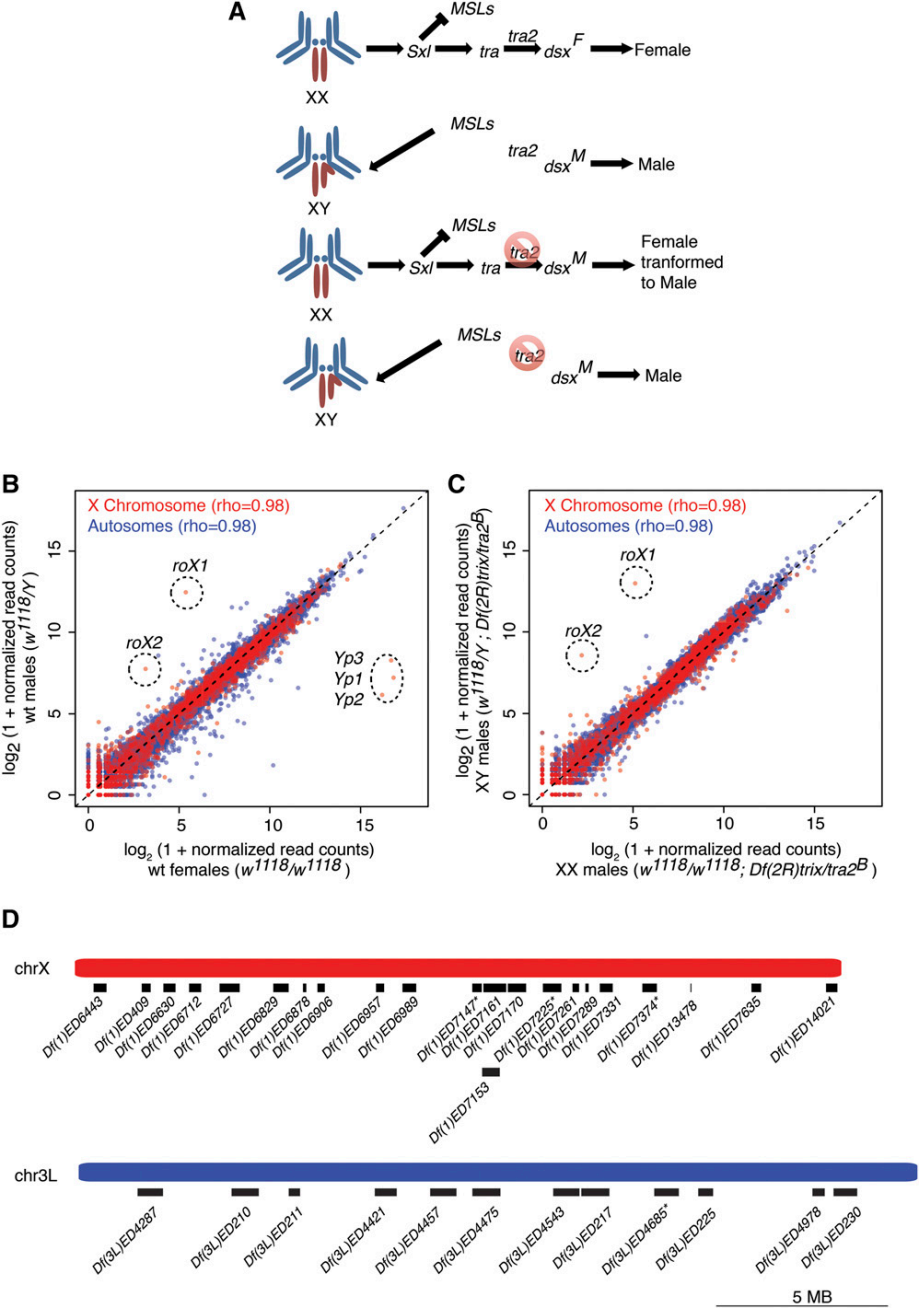
Gene expression in wild-type and sex-transformed heads. (A) Cartoon of sex determination (from top) in wild-type females with two X chromosomes (XX), wild-type males (XY), females transformed into males (XX), and control males for *tra2* (XY). Left panel shows sex chromosomes (red) and autosomes (blue). Major components of the sex determination hierarchy are *Sex-lethal* (*Sxl*), the MSL complex, *transformer* (*tra*), *transformer2* (*tra2*), and *doublesex* (*dsx*). *Dsx* encodes female (F) and male (M) transcripts due to alternative splicing mediated by *tra* and *tra2*. Mutations in *tra2* are indicted (red circle/slash). Gene expression scatterplots of wild-type XY male *vs.* XX female heads (B), or *tra2* mutant heads from XY males *vs.* XX females transformed into males (C), with genes and global correlations for X (red) and autosome (blue) indicated. Genes with the most dramatic gene expression differences are shown (dotted circles). (D) Cartoon of 22 X chromosome (red) and 12 autosomal (blue) *Dfs* used in this study, including three X chromosome and one autosomal *Dfs* (asterisk) excluded from analysis (see Table 1). Scale bar is as shown.

Sex-biased expression complicates the analysis of the subtle changes in gene expression between XX and XY flies. Therefore, we restricted our expression analysis to heads, because previous RNA-seq experiments revealed relatively limited sex-biased expression in this body part (Sturgill *et al.* 2013). As an additional measure to reduce sex-biased expression, we transformed XX females into somatic males (Figure 2A) using *transformer-2* alleles (*tra2^Df^/tra2^B^*) (Mattox and Baker 1991). When we compared the expression profiles of wild-type female (*w^1118^/w^1118^*) and male flies (*w^1118^/Y*) (Figure 2B), we observed a tight overall correlation between the head expression profiles. However, we did observe 670 genes with significantly female-biased and 602 with male-biased expression (FDR-corrected *P* < 0.05) in wild-type heads (Figure 2B, Supporting Information, File S1). When we compared gene expression in XX females transformed into males (*w^1118^/w^1118^*; *tra2^Df^/tra2^B^*) relative to XY males with tra2 mutations (*w^1118^/Y*; *tra2^Df^/tra2^B^*), we only observed 78 genes with significant XX-biased and 34 with XY-biased expression (Figure 2C, File S1). As expected, sex differences regulated upstream of *tra2*, such as expression of the dosage compensation transcripts encoded by *roX1* and *roX2*, remained XY-biased, whereas genes known to be regulated downstream of *tra2* (Salz and Erickson 2010), such as the three *Yolk protein* genes (Clough *et al.* 2014), were lowered to male levels in *XX*; *tra2^Df^/tra2^B^* flies (Figure 2, B and C).

### Gene expression by chromosome and gene dosage

If the X chromosome is inherently less or more sensitive to gene dosage reduction than autosomes, then we should observe differences between the dosage responses of chromosome X and 3L in XX females and in XX females transformed into males. To generate these hemizygous segments on the X and autosomes, we chose 12 deficiencies (*Dfs*) on chromosome 3L as controls and 22 *Dfs* on the X chromosome (Figure 2D). To avoid any potential bias associated with a particular chromosomal location, we selected a distribution along the length of the chromosome arms. We also avoided deletions reducing the dosage of genes known to be involved in dosage compensation or sex determination, because these could also confound our baseline measurements. Although it is possible that relatively small deletions show different dosage responses than whole chromosomes, we observed no correlation between *Df* extent and compensation level (not shown), as previously reported for the chromosome arm 2L (Malone *et al.* 2012).

**Figure 3:**
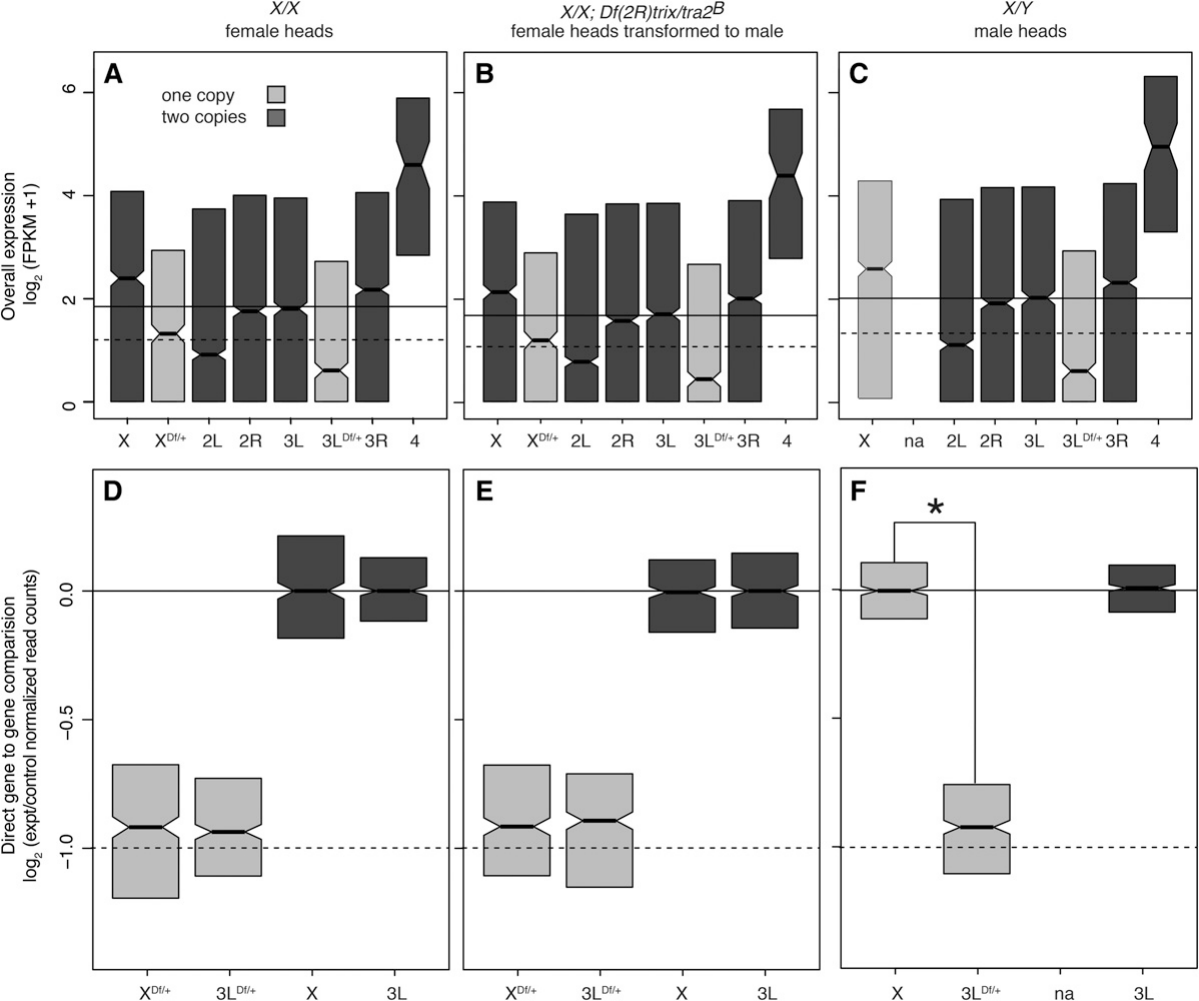
Dosage response boxplots. (A–C) Head gene expression values by chromosome arm, including a separate category for *Df/+* segments. (D–F) Ratiometric plots comparing the expression of genes in *Df/+* segments to the same genes in *+/+* (light). The controls (dark) were selected based on sampling the same number of genes as found in the experimental set. The *+/+* genes from *Df/+* flies were compared to the same *+/+* genes from *+/+* flies, or +/Y in the case of X-linked genes in XY males. (A, D) Females. (B, E) Females transformed into males. (C, F) Males. Plots show interquartile range (IQR; box), medians (bar), and confidence interval (±1.57 × IQR/√N).

We first looked at expression genome-wide in the samples parsed by chromosome arm with expression of hemizygous genes from *Df/+* chromosome regions binned separately (Figure 3, A–C). This analysis showed a strong bias for higher expression levels for chromosomes X and 4 genes in the regions of the genome with wild-type gene content. Chromosome 2L showed reduced overall expression in heads. This gene-content uncorrected analysis also showed essentially no compensation for X chromosome genes and a collapse in the expression of hemizygous genes on chromosome 3L. At face value, these observations show clear differences in baseline compensation between the X chromosome and the autosomes.

However, expression variance by linkage and by sampled region could also be due to differing chromosome and/or regional gene content as a confounding factor for analyzing dosage effects. For example, when we simply examined expression levels, median chromosome 2L expression was lower than expression hemizygous genes in *Df/+* regions on the X chromosome. These overall expression levels were essentially the reciprocal of the gene expression bias in reproductive tissues (Parisi *et al.* 2003; Sturgill *et al.* 2007), suggesting that different gene content, not dosage responses *per se*, are responsible. Specifically, there is under-representation of genes with male-biased expression in reproductive tissues on the X chromosome and fourth chromosome, and we show over-representation of genes expressed in heads on these chromosomes. Given that chromosome 4 is derived from an ancient X (Vicoso and Bachtrog 2013), these data suggest selection favors X linkage for genes expressed at high levels in heads. This is reminiscent of mammals where genes highly expressed in the brain are preferentially X chromosome–linked (Vallender and Lahn 2004). Reciprocally, there is an over-representation of genes with male-biased gene expression on chromosome 2L, and we show depletion for genes with high expression in heads on 2L. For our purpose here, sex-biased content is a confounding factor that hinders comparison of X chromosome expression levels with those of the autosomes.

Comparing the expression of the same genes when present in different copy number eliminates the effect of gene content differences (Figure 3, D–F). When we examined expression of a given gene when hemizygous, compared to the median expression of that gene in all lines (by bootstrapping), we observed a much more consistent dosage response, characterized by a strong decrease in median expression, with a median compensation level of ∼1.1-fold, in the case of both *Dfs* of chromosomes X and 3L. In both XX females and XX females transformed into males, the expression of hemizygous X chromosome and autosome genes was not significantly different, and there was not even evidence of a trend (Figure 3E). In XY males, X chromosome compensation was not significantly different from perfect, whereas compensation of autosomal hemizygosity was similar to that observed in XX fly heads (Figure 3F). These data indicate that the X chromosome has a generic response to decreased gene dosage that is similar to that of the autosomes.

## Discussion

*Drosophila melanogaster* sex chromosomes are thought to have arisen from an autosome pair and diverged to the current state by the gradual loss of genes on the nonrecombining Y chromosome (Vicoso and Charlesworth 2006). With the loss of more and more genes from Y chromosome, the chromosome-wide dosage compensation mechanism evolved as an effective machine for balancing X chromosome gene expression in flies with a highly degenerate Y chromosome. According to theory, the initial compensation for the one-dose genes on proto-X should be the same as the autosomal ancestor, which we suggest is the ∼1.1-fold upregulation we observed in this study. If the X chromosome became hemizygous little by little rather than all at once (Marin *et al.* 2000), then this might have provided time for selection to strengthened transcriptional regulatory interactions either before or during the emergence of the chromosome-wide compensation system. Additionally, the evolution of X chromosomes with a gradually degenerating Y chromosome should result in selection against chromosome-wide dosage compensation when the Y is young due to deleterious overexpression of genes with functional alleles on both the X and Y chromosomes. This shifting selection during the course of X chromosome evolution might result in strengthened compensation or selection for gene content that favors X-linkage for genes with more robust gene regulation. We found very little evidence for inherent differences between X and autosome compensation, suggesting that the extant X has the same inherent dosage response as an autosome. This is somewhat surprising given that the X chromosome shows a specialized chromatin structure in females and males (Zhang and Oliver 2010; Lee *et al.* 2014) and responds differently to gene dosage balance *in trans* (Sun *et al.* 2013), in addition to the evolutionary history outlined above.

The generic response observed for the X and autosomes is consistent with gene regulation playing a role in the response to copy number. For any given gene, we observed a significant correlation in compensation values among female, male, and sex-transformed flies bearing the same *Dfs* (not shown), supporting the idea that inherent gene-specific compensation levels contribute to the general dosage response (Malone *et al.* 2012). This includes evidence for additive effects of generic and chromosome-wide regulation (genes with the best compensation in Df/X flies tend to be overcompensated in XY flies), as previously reported in S2 cells (Zhang *et al.* 2010). We found median compensation (1.1-fold) at the low end of the previously reported range (1.1- to 1.8-fold) for partial compensation in *Drosophila* adults and cell lines (Johansson *et al.* 2007; Stenberg *et al.* 2009; McAnally and Yampolsky 2010; Zhang *et al.* 2010; Lundberg *et al.* 2012; Malone *et al.* 2012). Many of the differences in compensation values in these studies are due to low expression cutoff decisions. Increasing the cutoff values in the previous datasets also results in lower compensation values and decreasing the low expression cutoff values increased median compensation values in the dataset reported here (not shown). Thus, either there is better compensation of genes with low expression (Stenberg *et al.* 2009; Malone *et al.* 2012) or there is data compression due to expression noise and nonlinearity at low expression levels (Malone and Oliver 2011; Wang *et al.* 2014), or both. Additionally, reanalysis of cell line RNA-Seq datasets using more conservative low expression cutoffs, newer short-read aligners, and copy number callers (Boeva *et al.* 2012; Langmead and Salzberg 2012; Kim *et al.* 2013) showed consistent partial compensation ≤1.2-fold in most cell lines (H. Lee and B. Oliver, unpublished data). Human HeLa cells also show ∼1.2-fold compensation (Landry *et al.* 2013). However, even within a study with common methods and data handling, there are unexplained outliers (Lee *et al.* 2014). This highlights the importance of using carefully matched experimental and control biological samples and careful consideration of data handling in the analysis of dosage compensation. Given that X chromosome dosage compensation is very close to two-fold, whereas the effect of MSL mutations and gain-of-function are not, and given that the X does not appear to show boosted gene-by-gene compensation, then a substantial amount of X chromosome dosage compensation remains unexplained.

## Supplementary Material

Supporting Information
