# Supplementary material for: X Chromosome and Autosome Dosage Responses in Drosophila melanogaster Heads
Source: G3 (Bethesda). 2015 Apr 7;5(6):1057–63. doi: 10.1534/g3.115.017632 (PMC4478536; doi:10.1534/g3.115.017632)
Supplement: Supporting Information [file supp_5_6_1057__index.html]

X Chromosome and Autosome Dosage Responses in Drosophila melanogaster Heads — Supporting Information 

# X Chromosome and Autosome Dosage Responses in *Drosophila melanogaster* Heads

## Supporting Information for Chen and Oliver, 2015

**Files in this Data Supplement:**

- File S1 - Summary of differentially expressed genes between heads due to sex. (.xls, 225 KB)
